# Microbiomes: unifying animal and plant systems through the lens of community ecology theory

**DOI:** 10.3389/fmicb.2015.00869

**Published:** 2015-09-07

**Authors:** Natalie Christian, Briana K. Whitaker, Keith Clay

**Affiliations:** Evolution, Ecology and Behavior Program, Department of Biology, Indiana University, Bloomington, IN, USA

**Keywords:** community ecology, microbiome, bacteria, fungal endophyte, symbiosis, functional similarity

## Abstract

The field of microbiome research is arguably one of the fastest growing in biology. Bacteria feature prominently in studies on animal health, but fungi appear to be the more prominent functional symbionts for plants. Despite the similarities in the ecological organization and evolutionary importance of animal-bacterial and plant–fungal microbiomes, there is a general failure across disciplines to integrate the advances made in each system. Researchers studying bacterial symbionts in animals benefit from greater access to efficient sequencing pipelines and taxonomic reference databases, perhaps due to high medical and veterinary interest. However, researchers studying plant–fungal symbionts benefit from the relative tractability of fungi under laboratory conditions and ease of cultivation. Thus each system has strengths to offer, but both suffer from the lack of a common conceptual framework. We argue that community ecology best illuminates complex species interactions across space and time. In this synthesis we compare and contrast the animal-bacterial and plant–fungal microbiomes using six core theories in community ecology (i.e., succession, community assembly, metacommunities, multi-trophic interactions, disturbance, restoration). The examples and questions raised are meant to spark discussion amongst biologists and lead to the integration of these two systems, as well as more informative, manipulatory experiments on microbiomes research.

## Introduction

Communities, or species assemblages, are a fundamental unit of ecological organization, just as cell or tissue types are fundamental units of study for molecular biology and physiology. Originally developed for macroorganismal systems (Clements, 1916), many community ecology theories attempt to elucidate complex species interactions across space and time. Increasingly, these concepts are being applied to the study of some of our planet’s most complex and intimate communities—host-associated microbiomes. Both plants and animals are colonized by an astonishing number of symbiotic microbes (Raaijmakers et al., 2008; Zilber-Rosenberg and Rosenberg, 2008; Rodriguez et al., 2009; Lecuit and Eloit, 2013), and recent advances in sequencing technologies and data processing are finally affording researchers the opportunity to uncover the cryptic diversity and functions of these microbiomes (Zimmerman et al., 2014).

The microbiome can be made up of myriad prokaryotic and eukaryotic organisms, including bacteria, archaea, viruses, fungi, and protozoans. These groups of organisms have garnered attention due to their collective functional role in controlling host nutrition, metabolism, physiology, and immunology (Ottman et al., 2012). The importance of these different taxa can differ depending on the macroorganism with which they are associated. In animals, the bacterial microbiome significantly outnumbers and exerts more control over its host’s health and well-being than the fungal microbiome (Huffnagle and Noverr, 2013; Box 1). However, the opposite appears true for plants. Fungi, including leaf- and shoot-inhabiting fungi and root-associated mycorrhizae, appear to be the functionally prominent symbionts (Rodriguez et al., 2009; Porras-Alfaro and Bayman, 2011), despite being numerically less abundant than their bacterial counterparts (Lundberg et al., 2012; Bulgarelli et al., 2013, 2012). In particular, leaf- and shoot-inhabiting fungi (i.e., endophytes) are increasingly recognized for their impact on plant host health and utility in the study of community-level processes. Located in one of the most critical organs for energy processing (i.e., photosynthetically active leaves and shoots), these horizontally-transmitted endophytic fungi are readily culturable under laboratory settings and known colonizers of all plant species studied to date, including both wild plant species and model organisms such as *Arabidopsis*. Because endophytic fungi are easily cultured, they lend themselves well to studying the ecology of natural systems under controlled conditions in the laboratory. Although there are strong similarities in the ecological organization and functional significance of both animal-bacterial and aboveground plant-endophyte symbioses, the two systems historically have not been critically evaluated in conjunction with one another, potentially neglecting fruitful avenues for theoretical and experimental comparison. In this synthesis we highlight the commonalities of these two distinct research areas, which have had little communication between them, and outline how they may be unified by a common theoretical foundation.

Box 1 **Alternative Systems: Plant-Bacterial and Human-Fungal Microbiomes.**

Despite the functional prominence of the plant–fungal and animal-bacterial microbiomes, hosts are not exclusively colonized by one type of microbe—plants are riddled with bacteria, and animals may have 10^9^ resident fungal cells. For instance, the plant rhizosphere, the narrow zone of soil surrounding the plant root system, has a bacterial density of approximately 10^9^ cells per gram (Gans et al., 2005) and has been the subject of extensive research in the microbiomes field (Lundberg et al., 2012; Peiffer et al., 2013; Philippot et al., 2013). Similarly, the leaf surface may contain between 10^6^ and 10^7^ bacteria per square centimeter (Bulgarelli et al., 2013). Bacteria may colonize both the surface (“phyllosphere”) and the internal areas of leaf tissue (“endosphere”), with active habitat-switching occurring between the two (Beattie and Lindow, 1995). Bacterial diversity and community composition in the phyllosphere varies predictably across tissue and organ types (Leff et al., 2015) and over time (Shade et al., 2013). Similar to foliar endophytic fungi, these bacterial endophytes colonize the leaves from the air, neighboring plant tissue, plant debris (litter), or water (rain/flooding; Bulgarelli et al., 2013), and may play important roles in plant growth and development (Rosenblueth and Martínez-Romero, 2006; Ryan et al., 2008). A well-known example of a plant-associated bacterial symbiosis is the relationship between leguminous plants and root-associated rhizobia. In this mutualism, the plant supplies rhizobia with carbohydrates and various mineral nutrients, and in return the bacteria provides the plant with ammonia, which it synthesizes from atmospheric nitrogen (Kiers et al., 2003; Nelson and Sadowsky, 2015).

Although fungal constituents are considered part of the “rare human microbiome” and have traditionally been considered of minimal importance to human health, recent evidence suggests that these fungal residents play an underappreciated role in the regulation of human health. Fungi constitute a major component of both the fecal and skin non-bacterial, eukaryotic microbiota in humans (Parfrey et al., 2014). Some fungi appear to live commensally within their hosts during times of health, but transition into a pathogenic lifestyle upon disturbance of the bacterial microbiome. For instance, most human individuals are asymptomatically colonized by the fungus *Candida albicans*. However, perturbation of the microbiome (e.g., by an antibiotic), may facilitate an aggressive switch in this fungal species (i.e., a “bloom”), leading to dysbiosis and the onset of disease in host tissues (Huffnagle and Noverr, 2013). Physical and chemical interactions between fungi and bacteria are thought to influence the health and ecology of the collective oral microbiome (Krom et al., 2014). However, the more general roles of the myco-biome in host metabolism, immunity, and multi-trophic interaction with their bacterial neighbors remain largely unknown (Parfrey et al., 2014). Research cataloging the mycobiome in both sickness and in health will be important for medical scientists working to document and improve patient health.

The specific goals of this synthesis are twofold; first, to highlight the ecological and functional similarities between animal-bacterial and aboveground plant-endophyte microbiomes using six core theories in community ecology, specifically: successional theory, seed banks in community assembly theory, metacommunity theory, multi-trophic interactions, disturbance ecology, and restoration ecology (for a glossary of useful terms in community ecology, please refer to Table 1). These represent major theories in ecology that have been used independently and in conjunction for decades as a way to describe and predict macroorganismal abundance and distribution patterns. Our second goal is to show how these theories may be applied to animal and plant microbiomes and to further outline experiments that will spark research and discussion among a wide range of biologists (e.g., ecologists, animal scientists, agronomists, medical scientists, microbiologists). We do not intend this to be an exhaustive review of the current state of microbiome research nor that of community ecology, but rather to offer a novel perspective on the study of both using two exemplar systems.

**TABLE 1:** **Glossary of common terms used in community ecology**.

**Term**	**Definition**
Assemblages Commensalist	synonym for ecological communities a species that benefits from, but has no effect on the performance of another
Communities	a collective group of interacting species’ populations cohabitating within a defined local area
Facilitation	a species interaction where one species changes the environment in such a way as to improve the performance of another species
Functionally Redundant	describes communities that change in species composition following a disturbance, but the new community is functionally equivalent to the original
Horizontal Transmission	microbial dispersal between host individuals that occurs via the environment and not directly from parent to offspring
Host-Specificity	degree to which a microbial species is associated with a single host species or genotypes, or alternatively, is more widely associated with many different host species or genotypes
K-selected	describes species that have evolved strategies as strong competitors with lower reproductive effort and longer life spans
Latent Saprotrophy	a microbial lifestyle that involves a period of asymptomatic residence within plant tissues before switching to feeding on senescing, or dying, plant tissue
Mutualist	a species that benefits from, and also benefits the performance of another
Resilient	describes communities that change in species composition following a disturbance, but then quickly return to original species composition
Resistant	describes communities that do not change in species composition following a disturbance
r-selected	describes species that have evolved strategies for faster growth and reproduction, shorter life spans, and better dispersal and colonization across habitats
Trophic Level	a position in the food web that is determined by feeding mode and energy transfer, such as primary producer, consumer, or decomposer. A specific trophic level may contain many different species
Vertical Transmission	microbial dispersal between host individuals that is exclusively from parent to offspring, typically via eggs or seeds

## Defining Communities

The numeric diversity of microbial symbionts is astounding. Within the human-bacterial microbiome, up to 90–240 bacterial genera alone are associated with the skin (The Human Microbiome Project Consortium, 2012b), and at least 5 million non-redundant bacterial and phage genes are encompassed by the microbial community of the human gut (The Human Microbiome Project Consortium, 2012a). These communities are a product of at least 500 million years of coevolution between animals and their bacterial symbionts (Ley et al., 2008; McFall-Ngai et al., 2013). Similarly, evidence suggests that plants have played host to suites of aboveground fungal symbionts since their initial colonization of land 450 million years ago (Krings et al., 2007). Collectively known as “endophytes,” these fungal symbionts are increasingly recognized for their diversity and impact on host functioning (e.g., host physiology and immunology; Schulz and Boyle, 2005; Rodriguez et al., 2009). For the purposes of our paper, we define endophytic fungi (hereafter, “EF”) as those fungal symbionts that reside cryptically within healthy aboveground plant tissues such as leaves and shoots, form localized infections, and are horizontally transmitted amongst hosts (Rodriguez et al., 2009). This definition serves to distinguish EF from the vertically-transmitted systemic fungi typical of many grasses, as well as from bacterial endophytes, transient surface-dwelling microbes, and belowground fungal symbionts such as mycorrhizae, which form partially external symbioses with multiple host plants at the same time, and dark-septate root fungi that are generally restricted in their transmission from host-to-host by a structured soil medium (Box 2). The root-associated microbiome, known collectively as the rhizosphere, has been the subject of extensive research (Lundberg et al., 2012; Bulgarelli et al., 2013, 2012; Philippot et al., 2013; Box 1). However, increasingly researchers are using leaf -and shoot-associated fungal microbiomes (i.e., the “phyllosphere”) to move beyond characterization studies and into more manipulative exploration of the collective form and function of microbial communities in hosts.

Box 2 **Hereditary Symbiosis in Plants and Invertebrates.**

Vertical transmission of microbes through the germ line, combined with a systemic residence within the host, represents a unique lifestyle of certain symbionts in major groups of animals and plants. These one-to-one interactions between host and microbe include many classic examples in nature, such as the systemic, seed-transmitted fungal endophytes of cool-season grasses and morning glories (*sensu* “Class 1 Endophytes”; Rodriguez et al., 2009; Panaccione et al., 2014) and the symbiotic bacteria transmitted through the eggs of many invertebrates (e.g., *Wolbachia* of flies, *Buchnera* of aphids, *Rickettsia* of ticks; Oliver et al., 2014). Despite the prevalence of these invertebrate-symbiont interactions, the same strict co-evolution does not appear to exist in humans or other vertebrate animals, potentially in relation to the presence of both adaptive and innate immunity within vertebrates as opposed to the simpler invertebrate immune systems (Mcfall-Ngai, 2007). In plants, vertically-transmitted endophytes are known to induce a strong fitness benefit for many hosts, leading to the prevalence of this co-evolved mutualism among cool-season grasses in nature (Clay, 1988; Clay and Schardl, 2002). Despite, or perhaps because of, their relative simplicity, much more work has been done in these two systems on the co-evolution and ecology of host-symbiont interactions. Therefore, they represent a trove of useful information for studying their more hyper-diverse microbiome counterparts and should be incorporated into models of microbiome formation and function.

Leaf and shoot EF have been isolated from all plant species sampled to date, including aquatic and basal plant lineages (Bayman, 2006; Higgins et al., 2007; U’Ren et al., 2012; Sandberg et al., 2014). They are considered to be the most speciose and phylogenetically diverse members of the fungal kingdom (Arnold et al., 2000). Tens to hundreds of different fungal species may coexist within the foliage of a single host (Gamboa et al., 2002), where they may constitute up to 2.5% of photosynthetic biomass (Davey et al., 2009). Unlike most bacteria, which switch frequently between leaf surfaces and internal tissue, fungi maintain a more stable and intimate relationship with their plant hosts (Beattie and Lindow, 1995; Hallmann et al., 1997).

## Roles of the Microbiome Community

Just as free-living organisms provide extensive ecosystem services (e.g., pollination, nutrient cycling, water purification), microbial symbionts can significantly impact their surrounding host ecosystems. Although important defensive and nutritive roles are well-studied in the vertically-transmitted bacterial symbionts of insects and other invertebrates (Box 2), horizontally-transmitted bacterial symbionts of humans also manifest a variety of functional roles in their hosts and are now even considered analogous to an “organ” in and of itself (Lepage et al., 2013). The gut microbiome assists in the breakdown of dietary products and production of essential nutrients, such as vitamins B and D (Ley et al., 2008; Qin et al., 2010). Beyond their nutritional role, bacterial symbionts of vertebrates actively shape the mucosal layer of the small intestine and colon during development (Sommer and Bäckhed, 2013), which is later used as a selective barrier to reject pathogenic species (Hooper et al., 2012). Some gut bacteria (i.e., bifidobacteria) also take on a direct non-host immunity role by fermenting macronutrients into short-chain fatty acids as an energy source for host T-cells fighting off pathogenic bacterial blooms (Fukuda et al., 2011). Many other animal organs play host to bacterial symbionts (Box 3), including the skin (Chen and Tsao, 2013). In one study, mice grown without skin bacteria exhibited abnormal cytokine production and their T-cell populations were unable to mount an adequate immune response against the skin parasite *Leishmania major* (Naik et al., 2012). It is becoming increasingly clear that many human diseases are associated with an imbalance in the numerical composition or nutritive and immunological function of the microbiome, termed “dysbiosis.” The medical community now even recognizes the potential to use these shifts in bacterial abundance as a diagnostic tool to document and quantify disease severity (Hollister et al., 2014). A disrupted human microbiome has been linked to diverse pathologies, including kwashiorkor, a severe form of acute malnutrition (Smith et al., 2013); psoriasis (Statnikov et al., 2013); sexually-transmitted diseases (Brotman et al., 2012); and inflammatory bowel disease (Frank et al., 2007). A key role of the bacterial microbiota in carcinogenesis has also been proposed (Schwabe and Jobin, 2013).

Box 2 **Hosts as Landscapes: Spatial Variation in the Microbiome.**

Work on the human-bacterial microbiome has revealed distinct microbial communities associated with the gastrointestinal tract (gut), vagina, urogenital tract, oral cavity, nasal cavity, and skin, among other tissues and organs (Costello et al., 2009). Furthermore, even within these coarse delineations, evidence exists for finer-scaled intra-organ biogeography. For instance, compositional variation in skin bacterial communities has been identified along the right and left axes of the body and clustering also reveals distinctions among the head, arms, trunk, legs, and soles of the feet (Grice et al., 2009). This spatial variation appears to be driven both by the identity of the colonizing microbes themselves and habitat-specific factors such as whether the colonized organ is internal or exposed to air, moisture, and other vertebrate hosts (Grice and Segre, 2011; also see Metacommunity Theory). Other examples of intra-organ variation in bacterial communities include the oral cavity, where colonization patterns reflect the ability of each species to properly adhere to different surfaces such as tooth enamel, gingival tissues, or other bacteria (Kuramitsu et al., 2007). Work on the human digestive tract has also demonstrated changes in community structure traversing across the mouth, throat, stomach, colon, and into fecal waste (Stearns et al., 2011). Inter-organ variability in microbial colonization is not simply reflected by the identity of bacterial colonizers, but also by the microbial biomass across sites. With mammals, most of the microbial load is internal, and more specifically, within the gut (The Human Microbiome Project Consortium, 2012a; McFall-Ngai et al., 2013). In these highly colonized habitats, density-dependent selection may have heightened implications for microbial species distribution and abundance.

Similar intra-host biogeography has been demonstrated in the plant microbiome. The broadest distinction falls between below- and aboveground plant organs. Belowground symbionts include arbuscular- and ectomycorrhizal- fungi and root dark septate endophytes, as well as the bacterial colonizers that make up the bulk of microbial biomass in the rhizosphere (See Box 1). Belowground bacteria and fungi are fundamentally distinct from aboveground fungal endophytes in their ecology and host-to-host mode of environmental transmission (i.e., soil versus air and rain, respectively; Wearn et al., 2012). Aboveground EF communities are also known to exhibit organ specificity. For example, it has been shown that distinct endophyte communities are harbored in leaf, petiole, and stem tissues (Mishra et al., 2012). Xylem and bark tissues in woody species are known to contain distinct endophytic communities as well (Santamaria and Diez, 2005; Martín et al., 2013). Interestingly, recent research has even revealed that aphid-induced galls (tumor-like growths on plant tissue) can contain different EF constituents than the surrounding leaf and petiole tissues. Moreover, the EF profiles of each gall differed by species of aphid, despite sharing the same host plant (Lawson et al., 2014).

Similarly, numerous studies have documented that EF may confer pathogen resistance to their plant hosts (Arnold et al., 2003; Ganley et al., 2008; Lee et al., 2009). Mechanistically, this is thought to occur via direct secretion of antimicrobial substances, competitively “out-crowding” fungal pathogens for plant tissue habitat (Rodriguez Estrada et al., 2011), or priming the host plant’s immune system against future pathogen invasion (Alabouvette et al., 2009; Hartley et al., 2015). For example, EF render the palatability of leaves unpredictable to herbivores by increasing the spatial heterogeneity of the chemical landscape within host-plant tissues (Carroll, 1988; Herre et al., 2007). Several studies of temperate trees have found negative effects of EF on leafmining or galling insects (Wilson and Carroll, 1997; Lawson et al., 2014), including evidence suggesting that leafmining insects are more likely to lay eggs on leaves with lower endophyte densities (Wilson and Faeth, 2001). Moreover, endophytes may also strengthen plants’ innate pathogen and herbivore defenses; colonization by a single, common endophyte (i.e., *Colletotrichum tropicale*) induced the upregulation of over 100 different host genes in *Theobroma cacao*, including many related to chemical defense and the hardening of cell walls (Mejia et al., 2014). The anti-microbials secreted by EF are being investigated for their potential as medicinal therapies for human diseases and some forms of agricultural pest control, a field known as bioprospecting (Strobel and Daisy, 2003; Porras-Alfaro and Bayman, 2011). Beyond host defense, endophyte colonization has been implicated in conferring abiotic stress tolerance to hosts, often by altering plant physiology (e.g., hormonal manipulation, water consumption; Rodriguez et al., 2009, 2008). Inoculation by a single endophyte has been shown to reduce wilting in cacao under drought conditions (Bae et al., 2009), although other studies using the same host have shown that infection by a suite of endophytes increases water loss in host plants (Arnold and Engelbrecht, 2007). This suggests that some EF may be conditionally mutualistic, or that crowding, while beneficial for pathogen defense, can more quickly drain hosts of necessary resources. Under what conditions compositionally distinct microbiomes can either prevent disease or induce stress in their hosts are parallel questions for future research in both animal and plant hosts.

## Applying Community Ecology Theories to Microbiomes

The field of community ecology has its roots in studies of plant and animal communities from the 1920s and 1930s (Clements, 1916; Gleason, 1926; Elton, 1927). In its infancy, single and isolated theories were often used to delineate complex species interactions and make predictions about the abundance and distributions of species across space and time (Mittelbach, 2012). By the 1980s, however, a paradigm shift toward a more pluralistic and integrative approach began to take hold. This modern view of community ecology considers the multitude of processes that can regulate species diversity and abundance at the local as well as regional scales. The integration of theories has catapulted research of macroorganismal communities in the last 30 years and we believe the time is ripe to begin advancing the study of microorganismal communities, using a balance of theory and technology, in order to gain a fuller appreciation for the ecology of these unseen systems.

**FIGURE 1:**
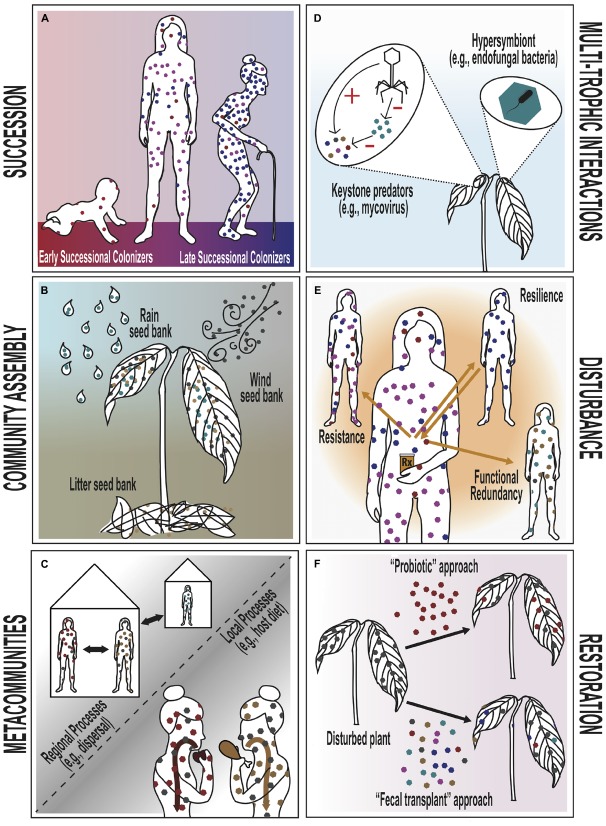
**Conceptual diagrams of six classical theories in community ecology, applied to the animal/human and plant microbiomes.** Each panel represents a separate theory. Hexagons are used throughout to represent the bacterial and fungal constituents of the human and plant microbiome, respectively. **(A)** Successional theory. In humans, late successional colonizers replace early successional colonizers. As succession proceeds, the density of the bacterial microbiome increases steadily, but diversity only increases into adulthood and declines thereafter in old age. **(B)** Community assembly theory. The primary sources of endophytic fungal colonizers for the plant microbiome are rain, wind and plant litter (e.g., leaves, twigs, bark). These spore sources can be considered akin to “seed banks” in community assembly theory. **(C)** Metacommunity theory. Human microbiomes can be influenced by both local processes, such as species interactions and habitat suitability (e.g., an individual’s diet), and regional processes (e.g., dispersal and extinction among or between households). The “local” and “regional” scales of microbiome communities may be defined flexibly (e.g., as organs, individuals, or households/populations). **(D)** Multi-trophic interactions. Within plants, endophytic fungi interact with many other organisms. Keystone predators and their analogs (e.g., mycoviruses) may suppress a dominant EF and indirectly promote a more diverse microbiome. Bacteria that reside intra-cellularly within fungal cells are known as “hypersymbionts” and may alter fungal behavior and have cascading health effects on the ultimate plant host. **(E)** Disturbance theory. Antibiotics represent an example of disturbance to the microbiome in humans. Four distinct types of microbiome community response to such a disturbance are theoretically possible: (1) microbiome composition remains unchanged (resistance), (2) microbiome composition changes but returns quickly to its original state (resilience), (3) microbiome composition changes but the new microbial constituents maintain the same function as the original community (functional redundancy), (4) or microbiome composition changes and does not retain original community function (not pictured). **(F)** Restoration ecology. A disturbed plant microbiome may be restored to its original composition or function through different approaches. In the “probiotic” approach, a plant is seeded with a single, presumably beneficial, EF species in order to restore the microbiome community, while the “fecal transplant” approach relies on inoculation by an entire healthy microbiome community in order to restore the target microbiome community.

At its core, the field of community ecology seeks to understand temporal and spatial dynamics of communities, interactions between a community’s component members, and the emergent properties of communities in response to change. We focus on six major theories in community ecology that are particularly relevant to testing for patterns of association and interaction within microbial communities: succession, community assembly, metacommunity dynamics, multi-trophic interactions, disturbance, and restoration (See Figure 1). While these six theories are not exhaustive, they are widely considered as cornerstone theories in studies of ecological communities (Begon et al., 2006; Mittelbach, 2012; Molles Jr, 2013) and have important application in the agricultural, medicinal, and environmental science fields. We conclude with a brief discussion of how these theories may be applied to the study of host-associated microbial communities versus the macroorganismal communities for which they were originally developed.

### Successional Theory

The theory of successional and temporal change in communities (i.e., predictable changes in species composition over time) has a long history in ecology (Connell and Slatyer, 1977). There is increasing evidence that the archetypal concept of successional transitions could inform changes in community composition and function for microbiomes (Figure 1A; Costello et al., 2012; Lozupone et al., 2012a). In human infants, as well as other mammals, primary succession of the microbiome begins prior to birth (Ottman et al., 2012). For example, in humans this consists of early successional microbial communities that are dominated by bifidobacteria (Hinde and Lewis, 2015). As we age, the microbiome community transitions into one with both higher numerical abundance and greater species diversity. Species diversity peaks in adulthood, with a microbiome dominated by *Bacteroidetes* and *Firmicutes*, but then declines (Koenig et al., 2011). By contrast, numerical abundance of the bacterial microbiome only increases with age (Koenig et al., 2011). Events such as disease onset, antibiotic treatments, and changes in diet can then cause non-random shifts in the composition of the microbiome (Koenig et al., 2011). Unlike higher animals, however, plants grow in size and develop new leaves and other organs continuously throughout their lives (Barthélémy and Caraglio, 2007; Klimesová and Klimeš, 2007; Box 3). This continual production of new habitat is denoted by increasing EF species diversity in individual leaves as they age (Arnold and Herre, 2003; Voříšková and Baldrian, 2013). Furthermore, leaf aging is thought to be associated with functional shifts in the roles of EF species from more mutualistic and commensalistic in mid-aged leaves to “latent saprotrophy” and decomposition acceleration in older leaves (Osorio and Stephan, 1991). The presence of such intra-host variation in age structure of habitats and communities within the plant–fungal microbiome presents itself as a unique opportunity to study temporal patterns of community assembly as the habitat (i.e., the leaf) ages and the host ages. To our knowledge no corollary exists for this concept in mammalian organs or tissues, including for skin microbiomes, making plants an excellent system in which to study microbiome succession while controlling for individual host variation and genetics.

One mechanism of succession occurs when early colonizers of a habitat facilitate the success of later colonizers (Connell and Slatyer, 1977). A transition in constituency from facultative to obligate anaerobes over the course of development is a common pattern seen in the fecal microbiomes of human infants (Sharon et al., 2013; Sommer and Bäckhed, 2013), as well as in bovine rumen microbiome (Jami et al., 2013). Facultative anaerobes are better able to colonize and tolerate these highly oxygenated and dynamic “virgin habitats,” but over time they engineer their environment to contain less oxygen and facilitate colonization by more stable obligate anaerobic communities. It is also well known that early colonizers of the human tooth microbiome, such as *Streptococcus oralis*, *Streptococcus gordonii*, and *Actinomyces oris*, perform the ecological role of adherence to the smooth and barren surface of the new teeth, subsequently acting as “bridges” and facilitators for secondary bacterial colonizers such as *Veillonella parvula* and *Porphyromonas gingivalis* as plaque formation proceeds (Kuramitsu et al., 2007; Kolenbrander, 2011). Successional transitions in microbiome constituency have also been documented in plant–fungal microbiomes, although there are far fewer examples of this ecological theory for EF (but see Martinson et al., 2012). In one study on two more pathogenic cousins of asymptomatic endophytes, primary residency by *Fusarium graminearum* increased the establishment success of *Fusarium verticillioides* in their mutual maize host (Picot et al., 2012). Another study documented facilitation or inhibition models of succession, which are contingent upon the order of arrival of pathogens and endophytes to a naïve environment. Secondary inoculation of EF can facilitate disease spread of *Pseudomonas* spp. in wild lima-bean hosts, but inhibit the disease-causing agent if inoculated first (Adame-Álvarez et al., 2014). This inhibition model, as well as other models of succession (i.e., “Tolerance”) are less explored than facilitation with regards to microbiomes.

Many experiments incorporating both traditional *in vitro* assays and advanced sequencing technologies, such as metagenomic and metatranscriptomic comparisons, could and should be used to test the components of successional theory as it applies to microbiomes in animals and plants. Late colonizing species may be stronger competitors (K-selected) in one-on-one *in vitro* assays or demonstrate higher population growth rates and tolerance in the face of external stress (e.g., antibiotics, fungicides, acidity, drought). Studies of microbial gene expression could reveal if early colonizers are faster growing (r-selected) or more tolerant of stressful conditions (e.g., oxidative stress; UV radiation; Lozupone et al., 2012b). Additionally, the question of whether it is possible to alter the trajectory of succession from healthy and stable to degraded and chaotic, or vice versa, has important implications for applied scientists studying long-term human health and plant fitness. If different successional trajectories can be engineered in animal and plant microbiomes, then medicinal and agricultural practices that induce stability should be promoted, while those practices that cause dysbiosis should be dissuaded (Lemon et al., 2012).

### “Seed Banks” and Community Assembly

Community assembly theory is used to understand the processes that shape the structure and function of communities and has been important in identifying patterns of macroorganismal communities across islands and continental landscapes. Given the horizontal mode of transmission of mammal-associated bacteria and leaf- and shoot-associated EF communities, community assembly theory may be particularly helpful in informing how microbiomes come together when their component members originate from different sources. In some special cases hosts directly facilitate the colonization of microbes from the environment. These cases have been studied in the squid-*Vibrio* model, where the host excretes substances to encourage *Vibrio fisherii* to colonize its light organs (McFall-Ngai et al., 2013) and leguminous plants that excrete substances to encourage colonization by rhizobia (Kiers et al., 2003; Nelson and Sadowsky, 2015; Box 1). However, for most other animals and plants, environmental transmission of microbial symbionts is much less directed. It is important to know the source populations, or “seed banks,” for horizontally-transmitted microbial species in the microbiome in order to make predictions about host health and function for animals and plants (Figure 1B; Harrison and Cornell, 2008). For example, it is known that the microbiomes of infants born via cesarean section (C-section) are fundamentally different from those born vaginally (Penders et al., 2006; Dominguez-Bello et al., 2010). Specifically, naturally-delivered infants are primarily colonized by the *Lactobacillus*-dominated vaginal microbiome, while C-section infants are more broadly colonized by non-lactobacillus-dominated bacteria from the surrounding environment, in particular the skin microbiome of attending physicians and the child’s mother. These differences in seed bank sources for C-section newborns have functional implications, and have been connected to susceptibility to methicillin-resistant *Staphylococcus aureus* (MRSA) skin infections (Dominguez-Bello et al., 2010). After birth, important source populations for the bacterial microbiome are related to life events, such as breastfeeding and the onset of a solid food diet (Matamoros et al., 2013; Bergström et al., 2014). Environmental surroundings such as the presence of a family dog (Azad et al., 2013) and types of fruits and vegetables consumed (Leff and Fierer, 2013), can also shape the assembly of a child’s microbiome community. One study has even demonstrated up to 14 bacterial types in common between children and the house dust from the home in which they were raised (Konya et al., 2014).

For EF communities, horizontal transmission from the environment is thought to be the predominant mode of dispersal (Rodriguez et al., 2009, but see Hodgson et al., 2014). Seedlings propagated in sterile environments (i.e., in growth chambers or greenhouses) have been shown to be virtually free of EF (Arnold and Herre, 2003), and studies artificially excluding air- and rain-borne spores from plants in the wild show reduced colonization by EF (Kaneko and Kaneko, 2004). In addition, insects are thought to ferry spores from plant to plant during feeding or pollination (Herrera et al., 2009), and neighborhood leaf litter is known to be an important source of fungal colonizers for aboveground leaf tissue (Kaneko and Kakishima, 2001; Herre et al., 2007). Some EF, such as *Coccomyces sinensis*, go on living within the leaf tissue even upon leaf abscission and decay (Koukol et al., 2011; Hirose et al., 2013) and thus are able to sporulate and re-colonize living tissues of their host or host’s offspring. Alternatively, leaf litter from one plant species could serve as a seed source for other plant species in the neighborhood, depending on the degree of host-specificity of the microbial symbiont. The functional and host-health consequences of these divergent sources of colonization for the plant microbiome could be important if, for example, the colonizers from litter are more often beneficial to their original host plant or host plant offspring. Alternatively, fungal seed banks from the air could represent cosmopolitan, or weedy, species with little benefit to the host they colonize.

By manipulating the relative contribution of different modes and sources of transmission for microbiome communities we can better understand how these communities differentially affect host functions. While this is a relatively straightforward pursuit in plant systems, it is ethically problematic to manipulate “seed” sources in humans. However, model organisms such as mice and flies could provide fruitful insights to human bacterial community assembly. It will be important in the future to alter and compare the effects of factors such as diet, kin, birthing mode, breastfeeding, and local environment in order to better understand inter-individual variation in microbiome community assembly (Costello et al., 2012), as well as host’s ability to facilitate colonization by specific beneficial microbes.

### Metacommunity Theory

Metacommunity theory is a relatively recent development in community ecology (Holyoak et al., 2005). The basic tenets of the theory posit that small-scale, local communities are interconnected at a regional scale via the processes of dispersal and extinction (Leibold et al., 2004). The specific species composition of the local communities is determined both by these two regional processes and the local processes of species interactions and habitat suitability (Holyoak et al., 2005; Leibold and McPeek, 2006; Mihaljevic, 2012). Metacommunity theory has been used to characterize communities of free-living macroorganisms, such as mosquito communities inhabiting water-filled tree cavities (Ellis et al., 2006), but researchers are increasingly advocating a role for metacommunity theory in studies of symbiotic microbes as well (Figure 1C; Costello et al., 2012; Mihaljevic, 2012). Symbiotic communities may even provide an advantage over the study of free-living communities in that “local” and “regional” scales can be defined flexibly, within nested levels of organization (Mihaljevic, 2012; O’Dwyer et al., 2012). For example, local microbiome communities can be defined at the organ level (e.g., gut, skin, leaf, stem), at the host level (e.g., individual person, plant), or even at the host population level (e.g., one family household, one forest). Locally, bacterial and fungal symbionts may compete for resources or avoid common enemies, which can influence community composition. But regionally, dispersal and extinction among these organs, hosts, or host populations can also influence microbiome communities across larger spatial scales. The flexible definition of local microbiome communities and nested organization of organs within hosts within host populations could be used as a tool to compare and contrast the importance of local and regional processes in a way not possible for free-living communities.

Despite this potential utility, few direct examples exist for the application of metacommunity theory to microbiome communities in either animals or plants. Indirectly, many studies have considered individual components of the theory, yet never under a unified framework. For example, the diet and societal role of the human host (e.g., hunters, gatherers, farmers) can be thought of as local processes that determine the quality and suitability of the gut habitat for different bacterial species in the gut microbiome. For instance, one study of traditional hunter-gatherer communities found a marked absence of *Bifidobacterium* relative to westernized urban controls, as well as strong differences in bacterial composition between the sexes, likely reflecting the sexual division of labor in this society (Schnorr et al., 2014). Similarly, EF colonization success and subsequent reproduction in plants has been shown to differ between shaded and full-sun leaf habitats, with increased prevalence of in *C. cladosporioides* and *A. alternata* for full-sun leaves in the same Japanese beech tree (Osono and Mori, 2003). Again, these patterns reflect the role of local habitat suitability processes in shaping the microbiome. Local species interactions have also been shown to shape the microbiome. In maize, interspecific interactions between EF species have frequently been detected. However, the outcome of these interactions, whether mutually beneficial or antagonistic, was dependent on the definition of “local community” used by the researchers (i.e., individual plant organs or entire plants, respectively; Pan and May, 2009). The regional process of dispersal is consistent with the higher degree of microbiome similarity among cohabiting family members (or plant individuals) than between families (or plant populations). For instance, research shows that the highest degree of MB similarity among family members exists for the skin microbiome (Song et al., 2013), suggesting that the regional process of dispersal is of greater relative importance in shaping the skin organ microbiome than the local metacommunity processes of species interactions or habitat suitability.

Future studies are needed to unite and compare the local and regional scale processes that shape the microbiome. Competition and dispersal assays could be performed *in vitro* for culturable symbionts, such as many EF, as a way to explain the absence, or differential abundance, of microbial species across hosts and host habitats. Efficacy of colonization success achieved by artificial inoculations could reveal how important the processes of host and organ specificity are to local community formation. Additionally, techniques such as molecular genotyping can be used to track specific microbial genotypes across space and time and quantify the patterns of dispersal and extinction for different microbiome species at a regional scale (Cockburn et al., 2013; McCormack et al., 2013).

### Multi-trophic Interactions

A major goal in ecology is to understand how multi-trophic level interactions (i.e., interactions amongst different groups and types of species) influence the diversity and abundance of species in communities. The ecology and evolution of symbiotic microbes is not just the simple byproduct of pairwise interactions between host and symbiont, but rather, is a function of complex multi-trophic interactions with predatory species, competing parasites, and other symbionts within the host, to name a few (Figure 1D; Agrawal et al., 2007). For instance, the population dynamics of many bacterial species and their specialist predatory phages cycle in classic predator-prey fashion over the course of human development (Sharon et al., 2013). In this case, viruses function analogously to the keystone predators in many food web networks because they control the population growth of otherwise dominant bacteria and maintain species diversity (Paine, 1966; Rodriguez-Valera et al., 2009). “Myco”-viruses have also been isolated from many fungi existing in plant species, including grapevines (Al Rwahnih et al., 2011), and chestnut trees (Springer et al., 2013), as well as corn and wheat (Chu et al., 2002). Mycoviruses have been implicated in reducing the virulence of fungal pathogens from elm and chestnut trees (Heiniger and Rigling, 1994; Buck and Brasier, 2001). However, whether mycoviruses control the population dynamics of more commensal or mutualistic fungal symbionts, such as EF, is a needed area for future research.

Other types of multi-trophic interactions are also important regulators of the microbiome in humans and plants. For example, recent work has uncovered the presence of endohyphal bacteria in the EF, *Pestalotiopsis* spp. This additional layer of symbiosis (i.e., hypersymbiosis) between the bacteria and its fungal host has been implicated in regulating hormonal transfer to the ultimate coniferous host, *Platycladus orientalis* (Hoffman et al., 2013). In mammals, studies have demonstrated that a high diversity of eukaryotic protists and fungi interact with bacteria during their residence within the gut (Parfrey et al., 2014) and that their direct contact with the bacterial microbiome can influence host health (Box 1). Intriguingly many of the factors thought to influence the abundance and distribution of the gut microbiome (e.g., host diet, age, environment) may also influence helminthic parasite communities. Helminth infection in pigs was followed by significant decreases in bacterial genes related to carbohydrate metabolism in the gut, but infection by another helminth species in mice models was correlated with increases in commensal bacterial species abundance (reviewed in Glendinning et al., 2014). Factorial experiments manipulating host diet (animals) or environmental resources (plants), different community members of the microbiome, and other trophic players could yield insights into the defensive or parasitic roles of various symbionts for their host. Such experiments could furthermore facilitate the design of therapeutic treatments of parasites using microbiome manipulation.

### Disturbance Ecology

Disturbance can be defined as a single disruptive event, or select set of events, that significantly changes community structure and function (Connell, 1978; Pickett, 2012). Ecological communities can respond in one of four ways to disturbance. Resistant communities experience no change in composition following the disturbance. Resilient assemblages change initially, but then return to their original state. Functionally redundant communities experience a change in species composition that has no impact on the overall community function. Finally, some communities are fundamentally altered by disturbance in both species composition and function (Allison and Martiny, 2008). Disturbances that disrupt the microbiome vary widely in magnitude and type (Figure 1E). Antibiotics, though largely considered one of modern medicines’ greatest advances, are being increasingly cited as an unprecedented type of disturbance to the human microbiome. Broad-spectrum antibiotics indiscriminately target bacterial wall components, thus eliminating many non-target commensalist and mutualistic microbes in addition to undesired bacterial pathogens (i.e., low community resistance; Lozupone et al., 2012a). Similarly, increasing the frequency of this form of antibiotic disturbance can lead to long-term shifts in the microbiome composition of healthy human adults (i.e., low community resilience; Dethlefsen and Relman, 2011). Gorilla populations from central Africa that face a higher degree of anthropogenic disturbance and habituation have compositionally distinct gut microbiomes from those populations that face less anthropogenic disturbance. Although the causal mechanism for these changes is unknown, the structural differences in gut microbiome profiles are correlated with functional changes in short-chain fatty acid and metabolite concentrations (Gomez et al., 2015).

Clearly the integration of disturbance theory in the animal microbiome remains incomplete. However, there is even less evidence documenting the effects of EF disturbance for plant health and performance. Preliminary evidence suggests that EF community composition exhibits little resistance to disturbance. Physical disturbances, such as hail storms, have been shown to decrease the diversity of foliar EF communities in the Brazilian plant *Coccoloba cereifera* (Fernandes et al., 2011). Other recent research shows startlingly long-term effects of physical disturbance for plant EF communities following the 2010 Deepwater Horizon Oil spill in the Gulf of Mexico. Characterization of these EF communities in smooth cordgrass showed near total loss of leaf EF, even 3 years after the original oil spill occurred (Kandalepas et al., 2015). In both of these situations, however, the functional consequences of reduced microbiome diversity remain unknown.

Future research is needed to characterize the response of host-associated microbiomes to diverse types of disturbance. This could be done by first manipulating disturbance frequency and/or intensity across a gradient and then measuring the subsequent changes in microbiome community composition and function. In humans and other animal systems, such efforts could improve our understanding of how certain pathologies are initiated in disturbed hosts. Likewise, empirical explorations of EF response to disturbance will be needed in order to make predictions about the effects of shifting anthropogenic activity and climate on plant stress tolerance and performance (Allison and Martiny, 2008; Porras-Alfaro and Bayman, 2011). Alternatively, understanding such functional consequences of microbiome disturbance could be used as a management technique for reducing the fitness of noxious or invasive plants or animals. For example, the EF microbiome of the invasive plant *Phragmites australis* (common reed) is currently being investigated with the ultimate intention of using disturbance as a method to perturb the microbiome and reduce this plant’s competitive ability (Kowalski et al., 2015).

### Restoration Ecology

Restoration of a disturbed ecological community to its former healthy state can only be undertaken with a deep understanding of both the biology of the species to be restored and the physical nature of the habitat itself (Bradshaw, 1996). As we continue to gain a better understanding of the factors that disrupt the composition and functioning of the microbiome in animals and plants, we are increasingly left with the problem of how to effectively restore microbiome function for the host following degradation or dysbiosis. For the human microbiome, this has led to the recent development of two methodologies in particular: fecal transplantation and over-the-counter probiotics (Figure 1F; Lemon et al., 2012). Although both restoration methods involve seeding presumed beneficial microbes into the existing host microbiome, they operate in fundamentally different ways. Fecal transplant, by literal definition, is a form of restoration in which the entirety of the gastrointestinal microbiota from a healthy host is seeded into an unhealthy individual experiencing dysbiosis (Kassam et al., 2013). The procedure has gained much public interest due to its high success rate at curing *Clostridium difficile* infections (CDI; Jorup-Rönström et al., 2012; Kassam et al., 2013). There are some indications that fecal transplants may also benefit patients suffering from a variety of other gastrointestinal and non-gastrointestinal conditions (reviewed in Aroniadis and Brandt, 2013), but these treatments are still in the early stages of testing (Ravel et al., 2014). In contrast, the probiotic approach typically involves seeding a single strain (e.g., *Lactobacillus* spp.), or limited number of strains, into the degraded microbiome of an unhealthy host. The goal of probiotic ingestion or application is for the symbiont(s) to either directly target pathogenic invaders using antagonistic secretions (Buffie et al., 2014), or indirectly facilitate the transition to more desirable microbiome metabolic functioning (Lemon et al., 2012). In one successful example, the use of a probiotic, *Lactobacillus delbrueckii*, to treat bacterial vaginosis syndrome in women was significantly more effective at long-term restoration toward a healthy vaginal microbiome than the traditional antibiotic treatment (Ling et al., 2013). Looking forward, we suggest that the narrow definitions of fecal transplants and probiotics in medicinal therapy could be applied to other forms of dysbiosis, representing methods of community restoration using high or low species diversity, respectively.

As an example of the probiotic restoration approach in the plant–fungal system, inoculation with the EF species *Trichoderma hamatum* promoted seedling growth and reduced wilt in cacao plants under drought conditions (Bae et al., 2009). Similarly, inoculation with a generalist foliar EF (*Alternaria* spp.) was shown to alter chemical secretions in its host forb, indirectly enhancing host competitive ability (Aschehoug et al., 2014, 2012). Preliminary evidence also suggests that inoculation with certain EF species can alter biomass production in forage crops (Kleczewski et al., 2012). Whole community seeding (the fecal transplant approach) is a less-explored concept in aboveground plant microbiomes. One example, however, showed that plant leaves artificially inoculated with a cocktail of seven EF were more resistant to pathogen infection than endophyte-free leaves (Arnold et al., 2003).

An important question to the future of both medicinal and agricultural therapies is which general method of restoration is the most appropriate for different states of dysbiosis, and will require careful consideration of the causes of dysbiosis and their pathologic outcomes. Inoculation studies with various levels of microbiome diversity (e.g., none, single species/low diversity, whole community/high diversity) could qualify whether and how host or microbiome community function is restored following pathogen invasion or other sources of disturbance. Furthermore, in order to assess whether these restoration techniques are more effective than traditional antibiotic or fungicide administration, a greater integration of molecular toolsets such as transcriptomics, metagenomics, and metabolomics will be needed to assign roles to the various states of “restored” microbiomes.

### Community Ecology Theory: Microbial vs. Macroorganismal Systems

Each of the six theories outlined here has provided important insights and directions for the study of macroorganismal communities. For example, successional theory predicts changes in forest communities through time (Halpern, 1989; Peterson and Pickett, 1995), while food web and multi-trophic interaction theories predict feeding relationships and patterns of energy transfer in aquatic communities (Wallace et al., 1997). However, community ecology theory has been less commonly used to understand the structure and function of microbial communities. By definition, host-associated microbiomes represent integrated communities occurring inside of a living host habitat. Therefore, the community ecology perspective may be especially relevant to microbiomes compared to other free-living microbial communities. The question remains, however, if such theories are more or less applicable to microbiomes than they are to macroorganismal communities. For example, the rapid generation times of microorganisms would likely accelerate the rate of community succession in microbiomes in comparison with macroorganismal communities. For similar reasons, microbiomes may be more resilient to external disturbances than macroorganismal communities, for which the impact of disturbance can persist for much longer. The local community assembly and metacommunity dynamics of both microbiomes and macroorganismal communities depend on the distribution, dispersal, dormancy, and extinction capabilities of their constituent species, as well as the local interactions amongst species and with their environment. More research is needed in order to determine whether microbial and macroorganismal communities differ fundamentally in the rate and magnitude of these processes. In contrast, the theory of multi-trophic interactions may be more pertinent to animal and plant macroorganismal systems due to their more complex food webs and patterns of energy transfer. In general, we do not know the relative applicability of each ecological theory to microbial and macroorganismal systems, or to human-bacterial and plant-EF systems specifically, but incorporating an ecological perspective into microbiome research will certainly help to answer this question.

## Future Directions in Microbiome Ecology

For upward of 30 years, a pluralistic approach to community ecology has helped explain complex species interactions of macroorganisms. The last decade has revolutionized our characterization of formerly “unseen” microbial communities, due to significant advancements in the quality and affordability of sequencing technologies and data processing. Paradoxically, although our datasets for microbial symbionts continue to expand, we are increasingly unable to interpret the findings, primarily due to a lack of basic ecological information for individual community members (Peay, 2014). Thus, despite technological improvements, and perhaps because of them, simple characterization studies are far more common than functional assays or manipulative experiments in animal and plant microbiome studies. Moreover, many regions of the microbial tree of life remain poorly described (Arnold et al., 2000; Kyrpides et al., 2014) and recognition of high genetic and functional diversity within and among microbial isolates is often ignored in the effort to assign workable taxonomic identities (Boon et al., 2014; Shapiro and Polz, 2014). In order to move the field of Microbiome Ecology forward, we need a conceptual shift that places value not only on describing what community members are present in the microbiome, but also on understanding what the ecological roles of these community members are. We must consider microbiome studies across multiple spatial, temporal, and trophic scales in order to better understand and predict community change. Likewise, identifying sources of degradation in these symbiotic communities, and implementing changes to restore them will be crucial if we are to make use of the knowledge gained from studying our microbial partners to improve human and animal health, agricultural productivity, and maintenance of healthy ecosystems. Essentially, we face the same challenges as animal, plant, and marine ecologists of the preceding century, except that the tools to measure and describe the “unseen majority” have only become available more recently. The unification of technological advances with community ecology theory could lead to both an increased breadth of described bacterial and fungal species, as well as more consistent predictions and understanding of their functional roles in nature.

In this synthesis we have defined and described lines of inquiry for core ecological theories established over the course of the previous century, as they may be used to delineate the functional significance of symbiotic microbes. Notably, the six community ecology theories described here are not mutually exclusive, nor exhaustive. Researchers should integrate and test different theories based on their system and experimental agendas. For instance, a conservation biologist could purposefully disrupt the microbiome of an invasive species using the principles of disturbance ecology, while simultaneously working to restore the microbiomes of native species and using the metacommunity framework to track microbial dispersal across habitats. On the other hand, a medical researcher might place greater emphasis on community assembly and successional theories as methods to predict which lifestyle factors alter microbial colonization in humans. Or similarly, how bacterial communities in the gut respond to multi-trophic interactions with viruses or other eukaryotes. In this way, disease onset as a result of microbial dysbiosis could be predicted or prevented. We believe that microbiome studies are at a critical turning point, moving from a simply descriptive phase into one that uses ecological principals and experimental manipulation to achieve better understanding and application. In order to move this emerging field forward, microbiome researchers and applied scientists alike must collaborate and communicate the theories and results of their respective fields. Community ecology should then be seen as fulfilling an important niche amongst a broad array of disciplines: a science developed to understand complex species interactions and make cross-system comparisons.

## Author Contributions

Both NC and BW equally drafted and edited this manuscript, with substantial editorial remarks from KC.

### Conflict of Interest Statement

The authors declare that the research was conducted in the absence of any commercial or financial relationships that could be construed as a potential conflict of interest.
